# P-1998. An assessment of appropriate use of methicillin-resistant Staphylococcus aureus nasal PCR testing

**DOI:** 10.1093/ofid/ofaf695.2162

**Published:** 2026-01-11

**Authors:** Mary Beth Marshall, Justin Zimmerman, Pramodini Kale-Pradhan, Leonard Johnson

**Affiliations:** Henry Ford St. John Hospital, Detroit, Michigan; Detroit Receiving Hospital, Detroit, Michigan; Wayne State University Eugene Applebaum College of Pharmacy and Health Sciences, Detroit, MI; Henry Ford St. John Hospital, Detroit, Michigan

## Abstract

**Background:**

In our institution, Methicillin-Resistant *Staphylococcus aureus* (MRSA) nasal polymerase chain reaction (PCR) testing is commonly performed in patients with healthcare associated pneumonia (HAP) to determine the need for anti-MRSA therapy. In addition, it can be used to assess for nasal MRSA colonization for pre-surgical screening that is less than 7 days prior to surgery. Nasal culture is performed to assess *Staphylococcus aureus* colonization in patients undergoing elective surgery in ≥ 7 days. The purpose of this project was to determine if the MRSA nasal screen PCR test and culture were being used appropriately.

**Methods:**

Patients who received either a MRSA nasal PCR test or MRSA nasal screen culture from October 2023 to January 2024 identified by the laboratory department were included.Nasal MRSA PCR test was deemed appropriate if used to determine the need for anti-MRSA therapy of HAP or used to assess nasal MRSA colonization for presurgical screening that is less than 7 days prior to surgery. Nasal culture was deemed appropriate if used to assess *Staphylococcus aureus* colonization in patients undergoing elective surgery in ≥ 7 days. Inappropriate use was defined as a indication other than pneumonia or presurgical screening.

**Results:**

Among the 191 patients who had MRSA testing, 60 were in the PCR and 131 in the culture groups respectively. Among the PCR group, the test was ordered appropriately in 58 of 60 (96.7%) including 60.3% for MRSA pneumonia and 39.7% for urgent surgeries. In contrast, only 60 of 131 (46%) with nasal cultures were appropriately ordered. In the nasal culture group, the incorrect test was ordered in 41% of the cases and 13% of this group had the incorrect indication. Overall, of the 191 cases, 118 (62%) had the correct test and indication; 54/191 (28%) had the incorrect test but correct indication; and 19 of 191 (10%) of the cases had the incorrect indication and therefore inappropriate use.

**Conclusion:**

PCR was used predominantly for the correct indication; however, the nasal culture was inappropriately used. Educational efforts are needed to raise awareness of appropriate use of nasal MRSA testing methods.

**Disclosures:**

All Authors: No reported disclosures

